# Natural Landscape, Infrastructure, and Health: The Physical Activity Implications of Urban Green Space Composition among the Elderly

**DOI:** 10.3390/ijerph16203986

**Published:** 2019-10-18

**Authors:** Carme Miralles-Guasch, Javier Dopico, Xavier Delclòs-Alió, Pablo Knobel, Oriol Marquet, Roser Maneja-Zaragoza, Jasper Schipperijn, Guillem Vich

**Affiliations:** 1 Geography Department and Institute of Environmental Science and Technology (ICTA), Autonomous University of Barcelona, Cerdanyola del Vallès, 08193 Barcelona, Spain; 2 Institute of Environmental Science and Technology (ICTA), Autonomous University of Barcelona, Cerdanyola del Vallès, 08193 Barcelona, Spain; javier.dopico@e-campus.uab.cat; 3 Institute of Urban and Regional Development, University of California, Berkeley, 94720-1820 CA, USA; xavidelclos@berkeley.edu; 4 Environment and Human Health Lab (EH2 Lab) and Institute of Environmental Sc +ience and Technology (ICTA), Autonomous University of Barcelona, Cerdanyola del Vallès, 08193 Barcelona, Spain; 5 ISGlobal (Barcelona Institute for Global Health), Cerdanyola del Vallès, 08193 Barcelona, Spain; oriol.marquet@isglobal.org; 6 Environment and Human Health Lab (EH2 Lab) and Institute of Environmental Science and Technology (ICTA), Autonomous University of Barcelona, Cerdanyola del Vallès, 08193 Barcelona, Spain; roser.maneja@uab.cat; 7 Research Unit for Active Living, Department of Sport Science and Clinical Biomechanics, University of Southern Denmark, Odense, 5230, Denmark; jschipperijn@health.sdu.dk; 8 Geography Department, Autonomous University of Barcelona, Cerdanyola del Vallès, 08193 Barcelona, Spain; guillem.vich@uab.cat

**Keywords:** urban green spaces, physical activity, seniors, landscape, Barcelona

## Abstract

Urban green spaces (UGS) have been linked with a series of benefits for the environment, and for the physical health and well-being of urban residents. This is of great importance in the context of the aging of modern societies. However, UGS have different forms and characteristics that can determine their utilization. Common elements in UGS such as the type of vegetation and the type of surface are surprisingly understudied in regard to their relationship with the type of activity undertaken in UGS. This paper aims to explore the relationship between landscape diversity and the type of surface with the time spent and the physical activity intensity performed by seniors. To do so, this study uses GPS tracking data in combination with accelerometer data gathered from 63 seniors residing in Barcelona, Spain. Results showed that senior participants spent little time inside the analyzed UGS and sedentary behaviors (SBs) were more common than physical activities (PAs). The presence of pavement surfaces positively influenced the total time spent in UGS while gravel surfaces were negatively associated with time spent in active behaviors. The provision of well-defined and maintained paved areas and paths are some key infrastructures to be considered when designing UGS for overall urban residents and, especially, when aiming to potentiate the access for senior visitors.

## 1. Introduction

The provision of Urban Green Spaces (UGS) has been noted as a key strategy for policy-makers towards the achievement of sustainable urban development and the improvement of health and well-being for urban residents [1,2]. Being in contact with nature in UGS has been shown, among many health benefits, to lessen physiological stress [3,4]; to boost social interactions [5]; and to mitigate air pollution, heat, and noise levels [6,7]. In addition, UGS have also been associated with increased physical activity (PA) levels [8], as natural environments act as a facilitator of healthy habits since they provide easy access to safe and engaging settings for practices such as walking or sports [9,10,11]. Both physical and mental health benefits linked to UGS are also fundamental for tackling the challenges of aging in modern societies [12]. The possibility to perform PAs in UGS has been associated with decreases in the incidence of cardiovascular diseases, morbidity, and chronic diseases and also with the improvement of functional capacity and cognition among the senior population [13,14].

Public parks and gardens are the most common and most researched types of UGS around the world [8,15]. Nevertheless, the characteristics and composition of these types of UGS can vary widely depending on landscape diversity and availability of facilities, which can influence the type of activities that can be undetaken and the derived benefits for health to be associated with a particular UGS. Facilities such as playgrounds or sports fields have been widely linked with increased levels of PA by UGS visitors [8,16,17]. However, less evidence exists regarding other characteristics of UGS such as the type of surface, which is of high importance for senior visitors when they are walking and being physically active [18]. Additionally, considering the main function of UGS which is offering visitors the possibility to experience vegetation and natural elements [19,20], few studies have focused on the link between the type of vegetation and the type of activities performed [21,22,23]. 

The presence of different vegetation and surface types can make UGS more or less attractive to be visited or to perform certain activities in, which can influence the health benefits associated with UGS. Nowadays there is a huge number of tools at hand for multi-dimensional quality appraisal of UGS [8]. Vegetated open areas such as grasslands or meadows act as recreational places where social interactions or sports activities may occur [24,25,26]. Likewise, the provision of shaded areas by way of trees has shown to positively impact walking through or sitting in these areas [27,28,29,30,31]. Further, not all types of shrubs are positive for safety, and only the low-lying shrubs would be acceptable [32]. 

The quality and quantity of facilities and amenities are also important characteristics that allow the performing of different types of activities [11]. However, a crucial and usually understudied characteristic especially relating to seniors involves the types of walkable surfaces in UGS. Well-defined and long pathways are basic facilities of UGS that are associated with increased PA levels among seniors compared to other areas such as grasslands [33]. Paved paths as well as walkable surfaces have proven to encourage activities such as walking, cycling, or dog-walking [11,34,35]. This is especially important among senior visitors, since they are sensitive to uneven surfaces, preferring to use paved paths due to their greater steadiness and sense of security [36]. 

The accessibility and size of UGS are other characteristics that may encourage or discourage visiting, improve the experience, and influence the type of activities being practiced. The distance from visitors’ homes to UGS is an important predictor for how often they use certain UGS, and closer distances have been associated with improved physical and mental health indicators among urban residents [37,38,39,40]. On the other hand, larger UGS have been linked with a higher availability of amenities, a better provision of facilities, a higher availability of space in which to be physically active, and increased PA levels [11,41].

In order to fill these research gaps, this study aimed to analyze the influence of vegetation and surface type on the PA of senior UGS visitors. Specifically, the analysis explores the effect of the type of vegetation and the different composition of walkable surfaces on the time spent in different physical intensity levels. To do so, GPS tracking data in combination with accelerometer data were used in this study to obtain the geocoded locations and PA intensity performed within a set of UGS in the city of Barcelona, Spain. 

## 2. Materials and Methods 

### 2.1. Study Area

Barcelona is a Spanish Mediterranean coastal city located in the northeast of the Iberian Peninsula (41°23’02’’, N 02°07’59’’ E). With an area of 102.16 km^2^, Barcelona lies between the confluence of the Llobregat and Besòs rivers to the southwest and northeast, respectively, and to the east of Collserola Natural Park [42,43]. 

The city contains 1135 ha of UGS in the form of parks and gardens, which equals to 7.1 m^2^ of green space per inhabitant [44]. This is a small area per person compared to other European cities with areas reaching 300 m^2^ per inhabitant in some cases, especially in the north of Europe [45]. These low levels of green space provision contrast with relatively high levels of greenness in squares and streets. In 2011, Barcelona had a ratio of street trees of 98.36 per 1000 inhabitants [46], surpassing the ratios in many Europe cities with the a ratio of street trees varying between 50 and 80 trees per 1000 inhabitants [47]. Of the vegetation present in UGS, 22.6% is indigenous, with a predominance of Quercus ilex, *Pinus halapensis* and *Platanus × acerifolia*, which account for 49% of urban trees in the city [42]. 

### 2.2. Participants and Study Design

A total of 269 participants were recruited from different senior centers between June 2016 and June 2017. All of Barcelona’s senior centers, both public (*n* = 21) and charity-run (*n* = 15), were contacted, from which nearly half acceded to participate (*n* = 14). The sample of senior centers was balanced between those located in high- and low-income neighborhoods. After explaining the conditions of the participation in each of the centers, an equitable participation of individuals in terms of gender was intended (153 females vs. 116 males). Participants were given an informative document about the project and they were asked to sign an informed consent form before being provided with an accelerometer that had to be worn on the wrist together with carrying a GPS logger for 7 consecutive days. The devices recorded their daily routes, locations, and PA levels, and were returned after 7 days. Participants were also asked to fill out a questionnaire about their sociodemographic profile, daily mobility, and PA habits together with their perceptions of their neighborhood. From the 269 initial participants we excluded 147 that lived outside the city boundaries and only retained the participants that had visited at least one UGS during the duration of the study (*n* = 63). For the purpose of this study, tracking and PA data provided by GPS devices and accelerometers were combined with publicly-available GIS data from Barcelona’s City Council to extract the use of UGS in Barcelona by senior residents. The study received the Autonomous University of Barcelona (UAB) institutional review board approval (CEEAH-3656).

### 2.3. Data Sources and Measurement

#### 2.3.1. GIS Data 

**Figure 1:**
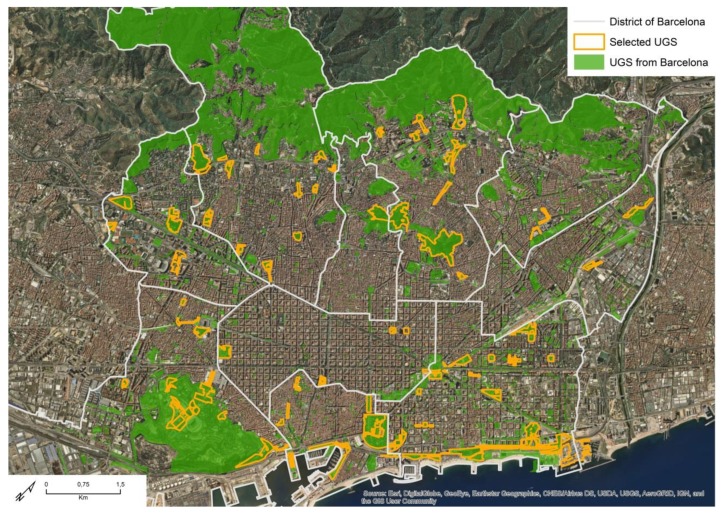
Distribution of UGS available in the city of Barcelona and the analyzed sample. Source: Own production based on ArcGIS^©^ Online base map [51].

We used the Land Use Map of Barcelona [48] to extract all the polygons categorized as Parks and Gardens (see Figure 1). Those polygons that were less than 10 meters away from each other were merged. Only areas that were accessible within the city’s urban continuum and were over one hectare in size were selected for this study. While there is no general agreement on a critical size threshold of UGS for specific health benefits [2], the selected threshold of one hectare fits within the threshold of international and national health recommendations (between 0.5 and 2 HA) [49,50]. All UGS meeting these criteria were visited to account for any differences between urban planning and the built reality. After the merging process 122 UGS were obtained. 

**Figure 2:**
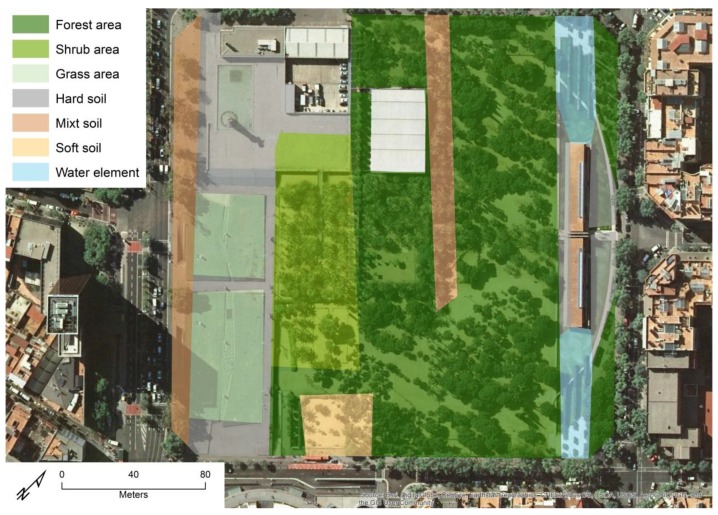
Example of the characterization of UGS by type of vegetation and walkable surface. Source: Own production based on ArcGIS^©^ Online base map [51].

The space inside these 122 UGS was divided and classified according to vegetation and surface type (See Figure 2). Vegetation diversity was identified by classifying areas within UGS as mostly provided with forest, shrubland, or grassland, while the different surface types in these UGS were divided into pavement, mix surfaces, or gravel soils. Water elements were also identified and classified. The field validations of the GIS data were performed by 8 field work technicians between April and May 2017. Finally, we filtered out all the UGS having no GPS tracking points from participants and kept the UGS that had attracted at least one visit. As can be seen in Table 1, within the final sample of 122 visited UGS, *forest* was the most represented vegetation type with 47.2% of the total area, followed by *pavement* with 16.4%, and *mix surfaces* with 14%. *Shrubland* and *grassland* were 6.7 and 6.8, respectively, with *water* (4.4%) and *gravel* (4.3%) being less abundant. 

#### 2.3.2. GPS Tracking and Accelerometer Data

The collected GPS tracking data reflected the position of participants in 15-second intervals based on a dynamic median accuracy of 2.9 m [52] thanks to the use of Qstarz Q-1000XT GPS loggers (Qstarz International Co., Ltd.,Taiwan, R.O.C.). Accelerometer data were collected using an ActiGraph GT3X (ActiGraph LLC, Pensacola, Florida USA) and its devices and for the present study were classified into two PA intensity categories: sedentary and active. Following Esliger et al. [53], a threshold of <216 vector magnitude (VM) counts per minute was used to define sedentary time and a threshold of 216 VM counts and more was classified as ‘active’. GPS and accelerometry data were merged using PALMS software [54] to couple spatial, temporal, and PA-related information. 

#### 2.3.3. Measurement of the Use of UGS 

**Figure 3:**
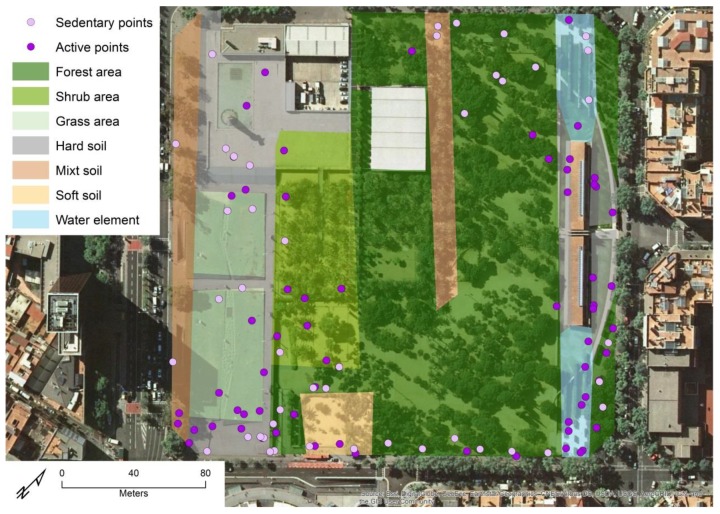
Example of GPS points of one participant in one of the selected UGS. Source: Own production based on ArcGIS^©^ Online base map [51].

An initial dataset with 5,423,327 GPS points was compiled by sampled participants during the data collection process. This number was reduced to 33,260 by eliminating all those points outside a UGS (see Figure 3). The dataset was reduced to 14,323 points after selecting the points of those participants who visited a UGS for at least 3 consecutive minutes [55]. Finally, since the devices were not waterproof, points registered in water areas have been removed from the database to avoid GPS accuracy errors. Thus, the final dataset contained 14,227 tracking points, which represented 290 visits by 63 users to 61 different UGS. 

### 2.4. Sample 

There were more male participants (55.6%) than female participants, and the share of participants between 65 and 75 years of age (54%) was higher than the older than 75 years of age (mean age of 81.1 years). Regarding health-related indicators, the number of participants perceiving their health as good (76.2%) prevails, and regarding their Body Mass Index (BMI), obese participants (76.2%) outnumbered those with a BMI <30. BMI was calculated using self-reported height and weight, and considering BMI’s over 30 as obese [56]. All participants were senior center (i.e., old-age community center) members.

#### Data Analysis

In order to analyze the use of the different UGS, the chosen outcome variables, for each participant, were *total time spent* in each UGS, *time spent in sedentary behavior,* and *time spent in active behavior*. Descriptive statistics display the different time expenditures by individual characteristics of participants and the characteristics of the analyzed UGS, as well as the use of different areas by both type of vegetation and surface within UGS by time and intensity. Due to the non-normal distribution of the sampled outcomes, the median was used in the descriptive statistics. Finally, a mixed-effects multilevel regression analysis for the 290 visits was used to test the association between log-transformed outcome variables and the explanatory variables which were grouped into socioeconomic personal variables and variables on the characteristics of UGS. The fixed effect analysis of these two models is aimed at measuring differences in each outcome variable between individuals after controlling for the independent explanatory variables at the individual level. The random effect explores the variation of the outcomes corresponding to the different profile and UGS-related factors at the level of UGS visit. 

## 3. Results

Participants spent a median of 8.5 minutes within the perimeter of the analyzed UGS (Table 2), and they spent a median of 6.5 minutes on sedentary behaviors (SBs) and only a median of 3.5 minutes on physical activities (PAs). Significant differences were found in the total time spent in the analyzed UGS between individuals with different BMI thresholds. *Obese* participants spent significantly less total time in UGS (8.0 median min; *p* = 0.02) and registered the shortest time on sedentary behaviors (5.3 median min; *p* = 0.022) than those with BMI < 30 (10.5 and 7.8 median min; *p* = 0.020 and *p* = 0.022 respectively). Regarding time on PAs, participants under 75 years of age registered significantly more time being active than older participants (3.9 and 2.0 median min. respectively; *p* = 0.003). Finally, significant differences were detected also regarding the *distance from home* when conducting physical activity in UGS, those participants who stood out were residing 301–600 meters away from the analyzed UGS (7.5 median min; *p* = 0.02). 

Table 3 summarizes time spent in each type of vegetated area and type of surface for all types of activities and by intensity. The results show a relationship between the area type and the time spent in the area. *Forest* was the most frequently found area type (29,046 m^2^, representing 47.2% of total UGS surface) being in turn the area were participants spent most of their total time (41.4%), their sedentary time (38%), and their active time (41%). A remarkable difference has also been detected in the case of *pavement* and *mix surfaces.* Participants spent higher shares of their total time (22.9% for *pavement* and 13.6% for *mix surfaces*), their sedentary time (24.5% and 14.2% respectively), and their active time (22.2% and 14.9%, respectively) than the shares these categories represent (16.4% for *pavement* and 4.3% for *mix surfaces*). This fact occurs also for *shrubland* areas although in a less pronounced way (6.8%, 9.4%, 9.8%, and 8.5%, respectively). On the other hand, total time and both sedentary and active time registered in *forest*, *grassland,* and *gravel* were lower than the share of land devoted to these areas.

Table 4 shows the multilevel regression analysis that explores the effect of both individual and UGS characteristics on total time, sedentary time and physically active time. This regression analysis shows how a share of variation in the different outcome variables (total, sedentary and active time) is attributable to differences between participants (intraclass coefficients of 6.03%, 18.73%, and 25.31%, respectively) and the proportion corresponding to the different explanatory factors explored at the individual level (19.16%, 8.34%, 52.21%, respectively). Overall, the chosen UGS factors can explain a small share of the total variance of the total time (1.36%) and sedentary time (1.70%), but a higher share in the case of active time (7.35%). Variation in UGS factors among participants was only found to be significant for sedentary behavior.

In relation to the variables affecting total time in UGS, only the proportion of *pavement* (B = 0.003) showed a significant effect on the total time spent in the analyzed UGS, indicating that the higher the proportion of this surface, the more time that is spent there by participants. Second, regarding sedentary time, *age* (B = 0.012) was the only significant factor explaining time spent at this intensity within UGS, showing that time spent sedentary increased with age. Third, *age* (B = −0.017), *BMI* (B = −0.042), and *gravel* (B = −0.019) showed a significant negative association with active time, while *distance from home* showed a positive association (B = 0.000). As *age*, *BMI,* and the *distance between UGS and the residence of participants* increased, less time was dedicated to physical activities. Finally, the *perceived health* of participants, the *total area of UGS*, the proportion of *forest*, *grassland*, *mix surfaces*, and *water* were characteristics that did not show a significant influence on any of the outcomes.

## 4. Discussion

This study explored the relationship between the provision of different vegetation types and walkable surfaces available and the type of activities performed and time spent in UGS by a group of seniors residing in Barcelona, Spain. Overall, this study has shown that senior participants spent short periods of time (a median of 8.5 minutes per visit) inside the analyzed UGS and they predominantly engaged in sedentary behavior during their visit. Moreover, areas designated as *forest* were where participants spent the majority of their total time but also sedentary time and active time, while *pavement* was the most used surface type for total time spent. 

Focusing on the characteristics of the analyzed UGS, the importance of walkable surfaces can be highlighted. On the one hand, *pavement* was associated with spending more time in the park. On the other hand, a higher proportion of *gravel* was associated with registering less active time by senior participants. Likewise, paths that are easy to walk along and without obstacles encourage outdoor activities among seniors and register the highest physical activity levels within UGS [25,33]. The importance of hard soils is more acceptable among senior users over another type of soil or environment with respect to the total time. Moreover, the significant effect of *distance from home*, also supported by previous authors [35,57,58], shows that shorter distances were associated with higher PA levels. 

The influence of different *types of vegetation* proved to be a non-significant factor for total, sedentary, and active time spent, which has also been found in some other studies [20,59]. Areas with higher proportions of *forest*, *shrubland,* and *grassland* corresponded with a similar sharing of time registered in these UGS in the descriptive statistics. The effects of the remaining analyzed explanatory factors overshadowed the influence of landscape diversity on the behavior of participants in UGS. The fact that the type of vegetation had no significant influence on the different outcomes does not mean it is not relevant. The type of vegetation could have been a decisive factor in the decision to visit specific UGS but did not encourage staying longer or increasing PA levels for senior visitors. Moreover, the *size* of the UGS was not significant when analyzed together with other explanatory factors, as it has also been seen in other studies [38].

Finally, the small amount of time registered in UGS might be explained by the age of the participants. Previous studies reported that seniors spent less time in UGS compared to other population groups, as most of the parks are geared towards serving youths or do not provide proximate, accessible, and safe spaces with well-maintained walking infrastructures [60]. Besides promenading or practicing sports, one of the main reasons for using UGS is spending time with friends [61,62]. The fact that 45.9% of the older population in Barcelona live alone [63] could be explained by the possible non-collective nature of walks registered by participants. Second, the predominance of sedentary behavior among senior participants has been confirmed by similar studies, corroborating seniors’ preferences for sitting on benches as the main reason for their sedentary behavior [55,64,65]. The links between age and sedentary behavior has also been confirmed [66] with *age* being a significant factor for both sedentary behavior and physical activity. In the present study, the increasing age of participants was associated with more sedentary time while younger participants registered significantly more time being physically active, as noted in similar previous studies [65,67]. Finally, another relevant observation in this study was that participants with a higher *BMI* spent less time being active, which is also in line with previous studies relating BMI with the use of the parks [68,69,70]. 

## 5. Limitations

This study is not without limitations. First, participants may have had a different walking behavior than usual as a result of being involved in this study. Likewise, another limitation is the fact that participants wore the accelerometer devices on their wrists, which means that certain activities might not have been registered because of the lack of movement by the arms. The analysis in this study might have been biased with participants engaging in this study presenting a better health condition than the average senior population. Correspondingly, the selection of participating senior centers was randomly selected and did not follow any pre-established sampling scheme. Additionally, other infrastructures of UGS that are important for seniors such as banks, drinking fountains, or toilets were not considered for this study. Future research should consider the influence of these elements, as well as the effect of the quality of the available infrastructure and the diversity of natural species on seniors’ behavior. The inclusion of motivations and preferences of seniors for using UGS should also be included in future studies.

## 6. Conclusions

This study aimed to investigate the impact of different types of vegetation and walkable surfaces in urban green spaces (UGS) on the utilization and intensity of activities performed by seniors. The importance of paved surfaces within UGS among seniors as was determined in this research adds to the debate around the unclear definition of green spaces, which might vary depending on the ecological conditions, the inherited built environment, or the political priorities, among others, of a particular urban context.

Based on the findings of this study, the surface type acquires paramount importance, especially when it comes to encouraging walking among seniors. Paved surfaces have proven to be crucial for spending more time in UGS while soft surfaces have shown to discourage physical activity. This study also showed that, when designing and planning UGS, not only are the quality and quantity of facilities in UGS important to improve the experience of visitors, but being cogniscient of the different profiles of potential users is also important. The predominance of sedentary behavior undertaken by this specific population group highlights the importance of considering the age of potential users when designing environments with the aim to be inclusive and accessible for everyone. Therefore, the provision of infrastructure in UGS should be oriented towards encouraging both sedentary behavior and physical activity in order to incentivize the improvement of both the physical and the mental health of their visitors.

Moreover, the important role of paved surfaces that was determined in this study can be useful for city planners when it comes to designing UGS adapted to senior population—which is of high relevance in a worldwide demographic context of aging of the population—both to attract more users and to incentivize them to stay for longer periods of time. 

## Figures and Tables

**Table 1:** Characteristics of the analyzed Urban Green Spaces.

Classification	Area	Definition	All UGS		Included UGS	
			m^2^	%	m^2^	%
Vegetation type	Forest	Area mostly occupied by trees	3,282,159	63.5	29,046	47.2
	Shrubland	Area mostly occupied by shrubs	215,230	4.2	4192	6.8
	Grassland	Area mostly compound of grass	878,604	17.0	4139	6.7
Surface type	Pavement	Surface mostly compound of cement or tailed soil	362,266	7.0	10,099	16.4
	Gravel	Surface compound of gravel and coarse sand	142,539	2.8	8625	14.0
	Mix surface	Complex areas with different types of surfaces	174,971	3.4	2651	4.3
Others	Water	Body of water such as fountain or pond	108,940	2.1	2723	4.4
Total area			5,164,710	100.0	61,475	100.0

**Table 2:** Descriptive statistics of median time spent and intensity by individual and UGS-related characteristics.

Characteristic	Categories	Total Time		Sedentary Time		Active Time	
		Min	*p*	Min	*p*	Min	*p*
Total		8.5		6.5		3.5	
**Individual Characteristic**							
Gender	Male	8.5	0.510	6.5	0.625	3.5	0.533
	Female	8.6		6.0		3.5	
Age	65–75 years-old	8.5	0.888	5.8	0.105	3.9	0.003 *
	>75 years-old	8.8		7.0		2.0	
Body Mass Index	Non-obese (<30)	10.5	0.020 *	7.8	0.022 *	3.8	0.672
	Obese (>30)	8.0		5.3		3.3	
Perceived health	Good	8.5	0.168	6.5	0.863	3.3	0.424
	Regular	7.6		5.8		3.8	
	Poor	14.4		6.9		4.4	
**Characteristic of the UGS**							
Distance from home	<300 m.	8.3	0.235	6.1	0.093	1.9	0.020 *
	301–600 m.	10.3		3.0		7.5	
	>601 m.	8.3		4.0		5.3	
Total area of UGS	<50,000 m²	8.5	0.963	6.3	0.112	3.1	0.738
	50,000–100,000 m²	8.3		6.3		4.3	
	>100,000 m²	8.9		7.2		1.4	

* Statistically significant results obtained from Kruskal–Wallis non-parametric one-way Analysis of Variance (ANOVA) or Wilcoxon Rank Sum test across individual and UGS-related variables.

**Table 3:** Descriptive statistics of the use of different areas within UGS by time and intensity.

Category	Area		Total time		Sedentary Time		Active Time	
	m^2^	%	Min	%	Min	%	Min	%
Forest	29,046	47.2	4.8	41.4	2,8	38.0	2.0	41.0
Shrubland	4192	6.8	5.3	9.4	4.5	9.8	1.7	8.5
Grassland	4139	6.7	1.1	2.8	1.8	2.4	0.5	2.7
Pavement	10,099	16.4	3.0	22.9	2.1	24.5	1,5	22.2
Mix surfaces	2651	4.3	4.1	13.6	3.3	14.2	2.3	14.9
Gravel	8625	14.0	3.5	10.0	3.2	11.1	1.4	10.7

**Table 4:** Mixed-effects linear regression ᵃ^,^ᵇ.

Fixed Effects	Total Time in UGS			Sedentary Time in UGS			Active Time in UGS		
	B	St. Error	*p*	B	St. Error	*p*	B	St. Error	*p*
**Intersect**	3.142	0.456	0.000	1.757	0.660	0.010	4.916	0.618	0.000
Gender (ref. male)	−0.022	0.069	0.753	−0090	0.099	0.367	−0.009	0.094	0.920
Age	0.001	0.004	0.719	0.012	0.006	0.049 *	−0.017	0.006	0.007 *
BMI (continuous)	−0.018	0.011	0.096	−0.007	0.015	0.630	−0.042	0.014	0.004 *
Perceived health (ref. good)	−0.055	0.084	0.515	−0.027	0.122	0.827	−0.073	0.114	0.526
Distance from home (meters)	0.000 **	0.000	0.349	0.000 **	0.000	0.958	0.000 **	0.000	0.027 *
Total area of UGS (m²)	0.000 **	0.000	0.594	0.000 **	0.000	0.503	0.000 **	0.000	0.610
Proportion of forest (%)	0.009	0.011	0368	0.022	0.014	0.110	−0.022	0.013	0.094
Proportion of shrub land (%)	0.002	0.003	0.473	0.004	0.004	0.384	−0.008	0.004	0.073
Proportion of grassland (%)	0.000 **	0.002	0.784	0.002	0.002	0.263	0.000 **	0.002	0.871
Proportion of pavement (%)	0.003	0.001	0.038 *	0.003	0.002	0.095	0.002	0.002	0.355
Proportion of mix surfaces (%)	−0.001	0.004	0.740	0.000 **	0.006	0.973	−0.002	0.005	0.705
Proportion of gravel (%)	0.002	0.007	0.829	0.016	0.009	0.084	−0.019	0.009	0.029 *
Proportion of water (%)	−2.140	1.837	0.246	−3.054	2.344	0.194	1.333	2.272	0.558
**Random Effects**	**B**	**St. Error**	***p***	**B**	**St. Error**	***p***	**B**	**St. Error**	***p***
Residual	0.169	0.015	0.000 *	0.214	0.021	0.000 *	0.169	0.015	0.000 *
Users	0.013	0.011	0.236	0.055	0.025	0.030 *	0.013	0.011	0.236

B: Coefficient estimate; St. err.: Standard error; t: *t*-value; *p*: *p*-value. * Significant value. ** Values with more than three decimals. ᵃ This model is based on the log-transformed dependent variable: total time in UGS (seconds), sedentary time (seconds), active time (seconds). ᵇ Intraclass coefficient (ICC) of total time: 0.06 (null model), 0.070 (full model). Proportion of the variance at Level 1 (19.16%) intraclass coefficient (ICC) of sedentary time: 0.187 (null model), 0.204 (full model). Proportion of the variance at Level 1 (1.70%) intraclass coefficient (ICC) of active time: 0.253 (null model), 0.139 (full model). Proportion of the variance at Level 1 (7.35%)

## References

[1] Kabisch N., Frantzeskaki N., Pauleit S., Naumann S., Davis M., Artmann M., Haase D., Knapp S., Korn H., Stadler J. (2016). Nature-based solutions to climate change mitigation and adaptation in urban areas and their rural surroundings. Ecol. Soc..

[2] WHO (2016). Urban Green Spaces and Health: A Review of the Evidence.

[3] Bratman G.N., Hamilton J.P., Daily G.C. (2012). The impacts of nature experience on human cognitive function and mental health. Ann. N. Y. Acad. Sci..

[4] Roe J., Thompson C., Aspinall P., Brewer M., Duff E., Miller D., Mitchell R., Clow A. (2013). Green space and stress: Evidence from cortisol measures in deprived urban communities. Int. J. Environ. Res. Public Health.

[5] Chiang Y.C., Li D. (2019). Metric or topological proximity? The associations among proximity to parks, the frequency of residents’ visits to parks, and perceived stress. Urban For. Urban Green..

[6] Livesley S.J., McPherson G.M., Calfapietra C. (2016). The urban forest and ecosystem services: Impacts on urban water, heat, and pollution cycles at the tree, street, and city scale. J. Environ. Qual..

[7] Margaritis E., Kang J. (2017). Relationship between green space-related morphology and noise pollution. Ecol. Indic..

[8] Koohsari B., Mavoa M.J., Villanueva S., Sugiyama K., Badland T., Kaczynski H., Giles-Corti A.T. (2015). Public open space, physical activity, urban design and public health: Concepts, methods and research agenda. Health Place.

[9] Akpinar A. (2016). How is quality of urban green spaces associated with physical activity and health?. Urban For. Urban Green..

[10] Romanillos T., Maneja R., Varga D., Badiella L., Boada M. (2018). Protected natural areas: In sickness and in health. Int. J. Environ. Res. Public Health.

[11] Schipperijn J., Bentsen P., Troelsen J., Toftager M., Stigsdotter U.K. (2013). Associations between physical activity and characteristics of urban green space. Urban For. Urban Green..

[12] Barnett D.W., Barnett A., Nathan A., van Cauwenberg J., Cerin E. (2017). Built environmental correlates of older adults’ total physical activity and walking: A systematic review and meta-analysis. Int. J. Behav. Nutr. Phys. Act..

[13] Heiland E.G., Welmer A.K., Wang R., Santoni G., Fratiglioni L., Qiu C. (2019). Cardiovascular Risk Factors and the Risk of Disability in Older Adults: Variation by Age and Functional Status. J. Am. Med. Dir. Assoc..

[14] Recchioni R., Marcheselli F., Antonicelli R., Mensa E., Lazzarini R., Procopio A.D., Olivieri F. (2017). Epigenetic effects of physical activity in elderly patients with cardiovascular disease. Exp. Gerontol..

[15] Vich G., Marquet O., Miralles-Guasch C. (2019). Green exposure of walking routes and residential areas using smartphone tracking data and GIS in a Mediterranean city. Urban For. Urban Green..

[16] Gomez L.F., Sarmiento R., Ordoñez M.F., Pardo C.F., de S.á T.H., Mallarino C.H., Miranda J.J., Mosquera J., Parra D.C., Reis R. (2015). Urban environment interventions linked to the promotion of physical activity: A mixed methods study applied to the urban context of Latin America. Soc. Sci. Med..

[17] Wu K.C., Song L.Y. (2017). A case for inclusive design: Analyzing the needs of those who frequent Taiwan’s urban parks. Appl. Ergon..

[18] Gaikwad A., Shinde K. (2019). Use of parks by older persons and perceived health benefits: A developing country context. Cities.

[19] Velarde M.D., Fry G., Tveit M. (2007). Health effects of viewing landscapes - Landscape types in environmental psychology. Urban For. Urban Green..

[20] Zhang W., Yang J., Ma L., Huang C. (2015). Factors affecting the use of urban green spaces for physical activities: Views of young urban residents in Beijing. Urban For. Urban Green..

[21] Aspinall P.A., Thompson C.W., Alves S., Sugiyama T., Brice R., Vickers A. (2010). Preference and relative importance for environmental attributes of neighbourhood open space in older people. Environ. Plan. B Plan. Des..

[22] Daniels B., Zaunbrecher B.S., Paas B., Ottermanns R., Ziefle M., Roß-Nickoll M. (2018). Assessment of urban green space structures and their quality from a multidimensional perspective. Sci. Total Environ..

[23] van Hecke L., Deforche B., van Dyck D., de Bourdeaudhuij I., Veitch J., van Cauwenberg J. (2016). Social and physical environmental factors influencing adolescents’ physical activity in urban public open spaces: A qualitative study using walk-along interviews. PLoS ONE.

[24] Arnberger A., Eder R. (2011). The influence of age on recreational trail preferences of urban green-space visitors: A discrete choice experiment with digitally calibrated images. J. Environ. Plan. Manag..

[25] Sugiyama T., Thompson C.W. (2008). Associations between characteristics of neighbourhood open space and older people’s walking. Urban For. Urban Green..

[26] Zhai Y., Baran P.K., Wu C. (2018). Spatial distributions and use patterns of user groups in urban forest parks: An examination utilizing GPS tracker. Urban For. Urban Green..

[27] Donovan G.H. (2017). Including public-health benefits of trees in urban-forestry decision making. Urban For. Urban Green..

[28] Jiang B., Chang C.Y., Sullivan W.C. (2014). A dose of nature: Tree cover, stress reduction, and gender differences. Landsc. Urban Plan..

[29] Jones B.A. (2019). Tree Shade, Temperature, and Human Health: Evidence from Invasive Species-induced Deforestation. Ecol. Econ..

[30] Vich G., Marquet O., Miralles-Guasch C. (2019). Green streetscape and walking: Exploring active mobility patterns in dense and compact cities. J. Transp. Heal..

[31] Wang R., Zhao J., Meitner M.J., Hu Y., Xu X. (2019). Characteristics of urban green spaces in relation to aesthetic preference and stress recovery. Urban For. Urban Green..

[32] Lindgren T., Nilsen M.R. (2012). Safety in residential areas. Tijdschr. Voor Econ. En Soc. Geogr..

[33] Besenyi G.M., Kaczynski A.T., Stanis S.A.W., Vaughan K.B. (2013). Demographic variations in observed energy expenditure across park activity areas. Prev. Med. (Baltim.).

[34] Arnberger A., Eder R. (2015). Are urban visitors’ general preferences for green-spaces similar to their preferences when seeking stress relief?. Urban For. Urban Green..

[35] Toftager M., Ekholm O., Schipperijn J., Stigsdotter U., Bentsen P., Grønbæk M., Randrup T.B., Kamper-Jørgensen F. (2011). Distance to Green Space and Physical Activity: A Danish National Representative Survey. J. Phys. Act. Heal..

[36] Rodiek S.D., Fried J.T. (2005). Access to the outdoors: Using photographic comparison to assess preferences of assisted living residents. Landsc. Urban Plan..

[37] Björk J., Albin M., Grahn P., Jacobsson H., Ardö J., Wadbro J., Östergren P.O., Skärbäck E. (2008). Recreational values of the natural environment in relation to neighbourhood satisfaction, physical activity, obesity and wellbeing. J. Epidemiol. Community Heal..

[38] Kaczynski A.T., Stanis S.A.W., Hastmann T.J., Besenyi G.M. (2011). Variations in observed park physical activity intensity level by gender, race, and age: Individual and joint effects. J. Phys. Act. Health.

[39] Duarte J.R., Cladera C.M. (2008). La localización intrametropolitana de las actividades de la información: Un análisis para la región metropolitana de Barcelona 1991–2001. Rev. Electrónica Geogr. Y Ciencias Soc..

[40] Nielsen T.S., Hansen K.B. (2007). Do green areas affect health? Results from a Danish survey on the use of green areas and health indicators. Heal. Place.

[41] Giles-Corti B., Broomhall M.H., Knuiman M., Collins C., Douglas K., Ng K., Lange A., Donovan R.J. (2005). Increasing walking: How important is distance to, attractiveness, and size of public open space?. Am. J. Prev. Med..

[42] Ajuntament de Barcelona (2018). Plan del Verde y de la Biodiversidad de Barcelona 2020. https://www.bcn.cat/estadistica/castella/dades/tpob/llars/padro/a2019/edat/t31.htm.

[43] Rojas C., Páez A., Barbosa O., Carrasco J. (2016). Accessibility to urban green spaces in Chilean cities using adaptive thresholds. J. Transp. Geogr..

[44] Ajuntament de Barcelona (2018). Statistical Yearbook of Barcelona City, 2018.

[45] Fuller R.A., Gaston K.J. (2009). The scaling of green space coverage in European cities. Biol. Lett..

[46] Ajuntament de Barcelona (2016). Statistical Yearbook of Barcelona City, 2016.

[47] Pauleit S.B.M., Jones N., Garcia-Martin G., Garcia-Valdecantos J.L., Rivière L.M., Vidal-Beaudet L. (2002). Tree establishment practice in towns and cities–Results from a European survey. Urban For. Urban Green..

[48] Ajuntament de Barcelona (2016). Land Use Map from Barcelona City Council. http://w20.bcn.cat/cartobcn/.

[49] Ambiente Italia Research Institute (2003). European Common Indicators: Towards a Local Sustainability Profile.

[50] Natural England (2010). Nature Nearby: Accessible Natural Greenspace Guidance.

[51] Esri, ‘World Imagery’ [basemap]. Scale Not Given. ‘World Imagery’. http://www.arcgis.com/home/item.html.

[52] Schipperijn J., Kerr J., Duncan S., Madsen T., Klinker C.D., Troelsen J. (2014). Dynamic accuracy of GPS receivers for use in health research: A novel method to assess GPS accuracy in real-world settings. Front. Public Heal..

[53] Esliger D.W., Rowlands A.V., Hurst T.L., Catt M., Murray P., Eston R.G. (2011). Validation of the GENEA accelerometer. Med. Sci. Sports Exerc..

[54] Jankowska M.M., Schipperijn J., Kerr J. (2015). A framework for using GPS data in physical activity and sedentary behavior studies. Exerc. Sport Sci. Rev..

[55] Thompson C.W. (2013). Activity, exercise and the planning and design of outdoor spaces. J. Environ. Psychol..

[56] WHO (2019). Mean Body Mass Index.

[57] Akpinar A., Cankurt M. (2017). How are characteristics of urban green space related to levels of physical activity: Examining the links. Indoor Built Environ..

[58] Pyky R., Neuvonen M., Kangas K., Ojala A., Lanki T., Borodulin K., Tyrväinen L. (2019). Individual and environmental factors associated with green exercise in urban and suburban areas. Heal. Place.

[59] Zhang S., Zhou W. (2018). Recreational visits to urban parks and factors affecting park visits: Evidence from geotagged social media data. Landsc. Urban Plan..

[60] Cohen D.A., Han B., Nagel C.J., Harnik P., McKenzie T.L., Evenson K.R., Marsh T., Williamson S., Vaughan C., Katta S. (2016). The First National Study of Neighborhood Parks: Implications for Physical Activity. Am. J. Prev. Med..

[61] Schetke S., Qureshi S., Lautenbach S., Kabisch N. (2016). What determines the use of urban green spaces in highly urbanized areas? - Examples from two fast growing Asian cities. Urban For. Urban Green..

[62] van den Berg M.M., van Poppel M., van Kamp I., Ruijsbroek A., Triguero-Mas M., Gidlow C., Nieuwenhuijsen M.J., Gražulevičiene R., van Mechelen W., Kruize H. (2019). Do Physical Activity, Social Cohesion, and Loneliness Mediate the Association Between Time Spent Visiting Green Space and Mental Health?. Environ. Behav..

[63] Ajuntament de Barcelona (2019). Statistical data from Barcelona City Council, 2019.

[64] Kaczynski A.T., Potwarka L.R., P B.E.S. (2008). Association of park size, distance, and features with physical activity in neighborhood parks. Am. J. Public Health.

[65] Sparling P.B., Howard B.J., Dunstan D.W., Owen N. (2015). Recommendations for physical activity in older adults. BMJ.

[66] O’donoghue G., Perchoux C., Mensah K., Lakerveld J., Van Der Ploeg H., Bernaards C., Chastin S.F., Simon C., O’gorman D., Nazare J.A. (2016). A systematic review of correlates of sedentary behaviour in adults aged 18-65 years: A socio-ecological approach. BMC Public Health.

[67] Knobel P., Dadvand P., Maneja-Zaragoza R. (2019). A systematic review of multi-dimensional quality assessment tools for urban green spaces. Health Place.

[68] Majeed F. (2015). Association of BMI with diet and physical activity of female medical students at the University of Dammam, Kingdom of Saudi Arabia. J. Taibah Univ. Med. Sci..

[69] Tolppanen A.M., Solomon A., Kulmala J., Kåreholt I., Ngandu T., Rusanen M., Laatikainen T., Soininen H., Kivipelto M. (2015). Leisure-time physical activity from mid- to late life, body mass index, and risk of dementia. Alzheimer’s Dement..

[70] Wang W.C., Worsley A., Cunningham E.G. (2009). Social ideological influences on food consumption, physical activity and BMI. Appetite.

